# 1891

**Published:** 1892-01-02

**Authors:** 


### 1891.

### At Berlin.

A year ago all the world was either at Berlin or looking in that direction. One of the greatest miracles the world has ever seen was said to have been wrought there by a German savant and physician—Dr. Robert Koch. Dr. Koch claimed that he had discovered a cure for consumption. The significance of such a discovery, if it had really been made, it is impossible to estimate. It would have ranked in importance with the discovery of the uses of steam and electricity in general science, and with that of the practical application of anæsthetics in medicine. Moreover, it would in all probability, have been the precursor of many applications of the same method of treatment.

But it is one thing to make a discovery, and quite another only to think you have made one. The idea of curing consumption by inoculation did not originate with Dr. Koch. Thousands of medical men, all the world over, were just as enthusiastic as he was to apply Jennerism or Pasteurism to consumption and other deadly maladies. The principles on which Koch proceeded were as familiar to them as to him; their professional ardour was as great, and their intelligence as keen. The world has heard nothing of those thousands, and why? Because they were possessed of that truly scientific temper which discriminates—a temper in which Koch proved himself so deficient at the critical moment. He could not wait; he could not judge himself; he rushed in with unscientific impetuosity; he risked all, and he lost all.

### Belated Pedants.

There are certain belated persons, even among English physicians, who profess to believe that some value may yet be found to be hidden in Koch's tuberculin. This need not surprise us. There are doctors, and intelligent doctors, who believe in homœopathy. As a cure for consumption, Koch's tuberculin has been universally condemned. Even as an aid to diagnosis it has been emphatically pronounced worthless, and nowhere more emphatically than at Berlin. From the first this was seen to be inevitable by persons who have a clear comprehension of the principles of human physiology. When Koch's original communication appeared in the *Deutsche Medizinische Wochenschrift*, on November 15th, 1890, we did not hesitate in this journal to express our conviction of the futility of the new treatment on purely physiological grounds. Still later, in the early part of 1891, and when the undignified *furore* at Berlin was at its noisiest, we repeated our condemnation in the most precise and definite terms.

It can be no gratification to any writer, least of all to a medical writer, to have to record the failure of high hopes in the region of scientific discovery and progress, and more especially in cases where human health and life are extensively concerned. But nothing is more injurious to the cause of true progress than an appearance of progress which has no reality about it. He who marches forward beyond the solid ground does but step on air, and precipitate himself into depths which are at least unknown and may be fatal.

Science, so far as the world is concerned, and even the medical world, is still in its infancy. The true scientific workers, the men of the Faraday stamp who are now living and at work, may almost be numbered on the fingers of one hand. But scientific dabblers are as numerous as flies in summer. It is with science, too, as it is in other departments of intellectual activity, the emptiest vessel yields the loudest sound. In these days, when war offers so few temptations to ambition, and the Church has no charms for the eloquent, science is the field into which clever and pushing young men rush by hundreds. They rush in, not because they are devoured by scientific enthusiasm, but because they wish to set all the world agape with wonder at their knowledge and their boldness, as Darwin, Tyndal, and Huxley have done. They do not see that those men were pioneers, and that as there can only be one Stanley in Darkest Africa, so a Huxley, a Tyndal, or a Darwin, though they may have imitators, can never have competitors on their own lines. Science is thrown open to the whole world now; and, though the world is in no sense scientific, yet it is rapidly taking the just measure of scientific men, and it is proving the truth of what it had long suspected, that they are exceedingly like other men, both in their greatness and in their littleness; and that unnatural marvels are no more to be expected from them than miracles are to be looked for at the hands of modern priests or untaught fanatics. It is an old world, and a sober. The sober temper brings us nearest to truth and right.

### Hypnotism Defunct and Unwept.

Hypnotism, which blazed forth like a prairie fire in 1890, has gone out very much like a prairie fire. It scarcely deserves so much as a passing word this year. Scientific and right-minded medical men are ashamed of all the fuss. It is but too evident that no amount of education, however varied it be, can turn a fool into a wise man. All the examinations of the past half century have failed to make the man of mere culture equal to the man of strong sense and natural intelligence. They will always fail. The world will never be turned upside down even to please schoolmasters and fill the pockets of "crammers." We are all finding this out now, and much to the world's advantage. Hypnotism has amused the foolish and employed the idle. It would be well if they could now find amusement of a more profitable kind.

### The Cholera Scare.

An alarm of cholera was raised in the early part of the year. It was feared that this deadly malady would follow upon the outbreak of influenza, as it has been known to do before. It was said also that cholera had been for some time unusually active in India, and that as it has generally travelled from east to west, so it would probably do again. But criticism and scepticism are now the order of the day, and Surgeon-General Sir William Moore, who has spent the greater part of his professional life in India, emphatically questions the received opinion that cholera travels from east to west. He thinks that it is just as probable that it travels from west to east. Moreover, he gravely doubts whether cholera is really a contagious disease at all. Sir William, who has probably had as large a personal experience of this disease as any living man, expresses the general conclusion that, given good air, good water, good food, and healthy life conditions all round, the populations of English towns need have little fear of cholera. This is distinctly a word of comfort, as well as a word of experience.

French Patriotism.

The French, not to be outdone by the Germans, have shown a patriotic determination during the year to discover a "consumption cure" of their own. In January it was announced that MM. Picq and Bertin had found in goats' blood a specific for tubercular disease. The goat, it was said, is the only animal known that is not liable to consumption. It was assumed that the cause was to be sought within the animal itself: and it was further assumed that the blood of the creature was its salvation. Goats' blood was therefore injected into consumptive patients; and cures were said to have resulted. It is now less than a year since MM. Picq and Bertin's announcements were made, and they are already ancient history—a blown bubble, and nothing more. Perhaps, by way of explanation of this French failure, it may be enough to say that the goat suffers from consumption like other animals when placed in appropriate circumstances, and that the immunity of the wild goat is, perhaps, not due at all to anything within himself, but to the pure air of the high and dry altitudes at which he, by preference, lives.

The Lords' Commission.

The Lords' Commission on hospitals got fairly into their work soon after the New Year commenced. It cannot be said that the Commission showed a want of patience. The noble lords gave the reins to a good many ladies, and the ladies showed their appreciation of the freedom both by variety and continuity of pace. They stepped out with a will, and contributed to the solution of the great hospital question facts both great and small. There were those who hinted that they sometimes contributed dreams, imaginations, and fancies, as well as facts. We will not here express an opinion. They certainly contributed a good deal of rhetoric, some of which tended rather to amusement than enlightenment. The Commission, like the loyal party voter at the poll, sat early and sat often. The members deserve all possible credit for their unwearied patience. Their report is not yet issued. Let us hope that when it appears it will be illuminated with the dry light of reason and fact, and that it will dissipate for ever the mists and vapours of ignorant criticism which have recently gathered about our noble medical charities.

An American Blunder.

Our American cousins made a movement in a new direction with regard to medical women during the year. The Governors of the Johns Hopkins University were offered a hundred thousand dollars if they would throw open their halls of learning to lady medical students on exactly the same conditions as to men. The golden calf was set up, and they fell down and worshipped it. We criticised their action at the time with severity; and many ladies, as well as men, both in America and in England, expressed entire agreement with the attitude we then took up. There has not yet been time to decide the merits of the question by actual experience, but we see no reason for departing from the principles and criticisms set forth at the time of the new departure.

Midwives' Registration.

An attempt was made by certain philanthropic members of Parliament and others to pass a Midwives Registration Bill through the House of Commons during the session. The object of the Bill was to provide that every woman who practised as a midwife should have some proper qualification for her work. It is difficult to imagine that anyone born and bred in a civilised country could object to such a measure. There are tens of thousands of poor women in England who are compelled to choose midwives to attend them in confinement, because they cannot afford to pay the moderate fee of even the humblest medical man. The State sought to insist that midwives should know something about their business. But the measure was compulsorily withdrawn. The trades union spirit among the more reckless class of medical men overcame every scruple of decency or humanity, and killed the Bill. *The Lancet* and the *British Medical Journal*, to their honour be it said, supported, and still support the principle of the measure. The recent election of direct representatives to the General Medical Council, shows that the medical opposition to the principle of midwives' registration, is weak both in numbers and in influence, and strong only in turbulence and denunciation. There can be little doubt that when a well-considered measure is again introduced to Parliament, it will have the support of all the most humane and intelligent members of the medical profession.

The Eastern Hospital.

"A Hospital Scandal," so-called, occurred in the Eastern Poor Law Infirmary, and engaged a great deal of public attention during the year. That scandal does not particularly concern us, because the Eastern Hospital is not really a "hospital" at all, but a Poor Law Infirmary. It is, in fact, an adjunct to, or rather an essential part of the workhouse. It has no more to do with the general and special hospitals of London than has a workhouse or a pauper asylum. It would not be necessary to refer to this question at all here, but that certain persons in public positions have chosen, either from ignorance or from wilfulness, to treat the affairs of the Eastern Poor Law Infirmary as if they were a part of our voluntary hospital system. Those persons should, in future, make themselves acquainted with at least the elements of a subject before they hasten to instruct the public thereupon.

Levites, not Good Samaritans, at Oxford.

It is with regret we chronicle the fact that the affairs of the Radcliffe Infirmary at Oxford remain *in statu quo*. On April 11th, 1891, our special commissioner wrote as follows: "To bring the Radcliffe Infirmary up to a level with other county institutions in this country the following alterations are imperatively called for: The front block should be entirely reconstructed or built from the foundation, and new fittings should be supplied to every portion of the building, ward sinks, baths, and other appliances all needing renewal. The wards situated in the out-lying blocks, if the fittings are modernised, may be left as they are, provided an adequate regard is shown for internal appearances in the way of repainting and renewal. A properly constructed nurses' home is a *sine quâ non*, and until this is forthcoming those responsible for the administration of the Radcliffe Infirmary cannot be held to adequately realise their responsibilities or to discharge them. More personal interest on the part of the inhabitants of Oxford in all that concerns the internal arrangement and management of the infirmary must be excited. It is perfectly evident that there is a lamentable absence of individual energy and continuous effort to maintain this institution in a high state of efficiency. These things ought not to be." The Managers at Oxford at first repudiated these criticisms, and then admitted their justice and force. Several meetings of the committee were held, and it was finally resolved to spend £10,000 on the necessary alterations. But though that resolution was passed more than six months ago, there has not been taken, so far as we are aware, one single practical step towards its realisation. Everybody knows how strong Oxford has long been in the polemics of its own peculiar form of Christianity; and everybody will now see how very weak that ancient University is in the practice of the merest rudiments of its own faith. *O tempora, O mores.*

THE PRINCESS OF WALES AND THE SECOND THOUSAND.

On July 24th the Prince and Princess of Wales received the second thousand nurses, who had joined the Royal National Pension Fund, at Marlborough House. The Prince, in a brief speech, assured the nurses that her Royal Highness the Princess "takes the deepest personal interest in this most beneficent society." The day was beautiful, and the nurses and invited guests were in the highest spirits. Her Royal Highness, unfortunately, was the victim of a severe headache. But, notwithstanding this, she took up her appointed place at the appointed time, and distributed to hundred after hundred of the nurses the certificates which constituted them members of the Royal National Pension Fund. The Princess has some of the characteristics of the famous Iron Duke of our national history. When she assumes the command of a great movement she makes up her mind that that movement is to be certainly victorious. She has made the Pension Fund not only successful but famous. All the civilised world is familiar with its history; and the United States of America have been so fired by generous emulation that they have resolved to have a Nurses' Pension Fund of their own. How well this National Pension Fund illustrates the principle that an intelligent purpose and unyielding perseverance command success and recognition all the world over! The nurses of the Pension Fund, on their part, have shown in many ways how gratefully they appreciate the leadership and gracious countenance of their Royal Highnesses the Princess and the Prince of Wales. They have united together in their hundreds to present to the Princess a beautifully carved screen of white wood, with their portraits, as a lasting tribute of their gratitude and loyal affection. The Princess has sent to each and all of them a letter of pleased thanks. Thus the nurses, too, in their turn, have as a permanent possession a charming memento of a happy and hopeful year.

EPILOGUE.

We have left ourselves but little space for the consideration of many important events in the hospital and medical year, such, for example, as the report of the Hyderabad Chloroform Commission, M. Lannelongue's chloride of zinc injections for tuberculosis, the Roussel treatment of phthisis, the foolish and unscrupulous attacks made upon the London Hospital, the progress of bacteriological knowledge, and other like questions. But what has been said indicates with convincing clearness the alertness and diligent thoroughness of physicians, surgeons, nurses, hospitals, and every department of medical science and practice. Whatever may have been their successes or their failures during the year, there can be no doubt of the strenuousness of their efforts and the worthiness of their aims. Medicine is no longer hide-bound by tradition and manacled by pedantic ignorance. It is alive, it is active, it is intelligent, it is courageous; and it strives with all its powers, and with much success, to play a great and noble part in a great and progressive civilisation.

